# Myocardial infarction, symptomatic third degree atrioventricular block and pulmonary embolism caused by thalidomide: a case report

**DOI:** 10.1186/s12872-015-0164-4

**Published:** 2015-12-18

**Authors:** Shengyu Zhang, Jing Yang, Xiaofeng Jin, Shuyang Zhang

**Affiliations:** Department of Internal Medicine, Peking Union Medical College Hospital, Chinese Academy of Medical Sciences, Beijing, China; Department of Cardiology, Peking Union Medical College Hospital, Chinese Academy of Medical Sciences, Beijing, China

**Keywords:** Thalidomide, Myocardial infarction, Third degree atrioventricular block, Pulmonary embolism

## Abstract

**Background:**

Thalidomide has been reported to cause numerous thromboembolic events. Deep vein thrombosis and pulmonary embolism are more common. It can also cause bradycardia and even total atrioventricular block. Rarely, it causes coronary artery spasm and even myocardial infarction. But almost simultaneous onset of myocardial infarction, third degree atrioventricular block and pulmonary embolism in one patient has not been reported so far.

**Case presentation:**

A 53-year old man presented because of chest pain, nausea and then syncope for several minutes. Previous medical history included neurodermitis for which thalidomide was given and hypercholesterolemia with simvastatin taking. The patient didn’t exhibit any other established risk factors for coronary artery disease. Electrocardiography showed sinus rhythm with third degree atrioventricular block and complete right bundle branch block, and precordial leads ST segment elevation. The diagnosis of acute coronary syndrome was suspected, but further coronary angiography demonstrated no flow-limiting lesions in coronary arteries, and temporary pacemaker was implanted. After admission, low SpO_2_ and elevated D-dimer level was mentioned. Further computed tomography pulmonary angiography revealed pulmonary embolism. Thalidomide was thought to be the cause of hypercoagulability and coronary spasm, so it was ceased immediately. Therapeutic low molecule weight heparin was initiated and then switched to warfarin with appropriate INR, and nifedipine was described for coronary spasm. The patient’s symptoms completely relived and SpO_2_ recovered, and atrioventricular block had disappeared during hospitalization with pacemaker removed.

**Conclusion:**

This is the very first case in which myocardial infarction, third degree atrioventricular block and pulmonary embolism almost simultaneously developed. We should be ware that anti-thrombotic prophylaxis, which needs further investigation for optimal drug and dosage, may be beneficial in thalidomide therapy. And it is also important to monitor patients taking thalidomide for signs and symptoms of bradycardia or higher degree atrioventricular block.

## Background

Oral immunomodulatory drugs, namely thalidomide and its analogues (lenalidomide and pomalidomide), presently play a crucial role in the treatment of multiple myeloma (MM) and other diseases such as Crohn’s disease, refractory aphthous ulcer in HIV and dermatologic conditions [1]. As it is cautiously being reintroduced into clinical use, new adverse effects are being described. Trials have reported numerous thromboembolic events attributed to thalidomide therapy, especially in multiple myeloma patients; deep vein thrombosis (DVT) and pulmonary embolism (PE) are more common, but occasional arterial thrombotic events have also been reported [1]; it also can cause bradycardia [2–4] and even total atrioventricular block (AVB) (the only case ever reported) [5]. Even Rarely, thalidomide can caused coronary artery spasm [5] and myocardial infarction (MI).

Herein we report the first case taking thalidomide with almost simultaneous onset of MI, third degree AVB and PE.

## Case presentation

**Fig. 1:**
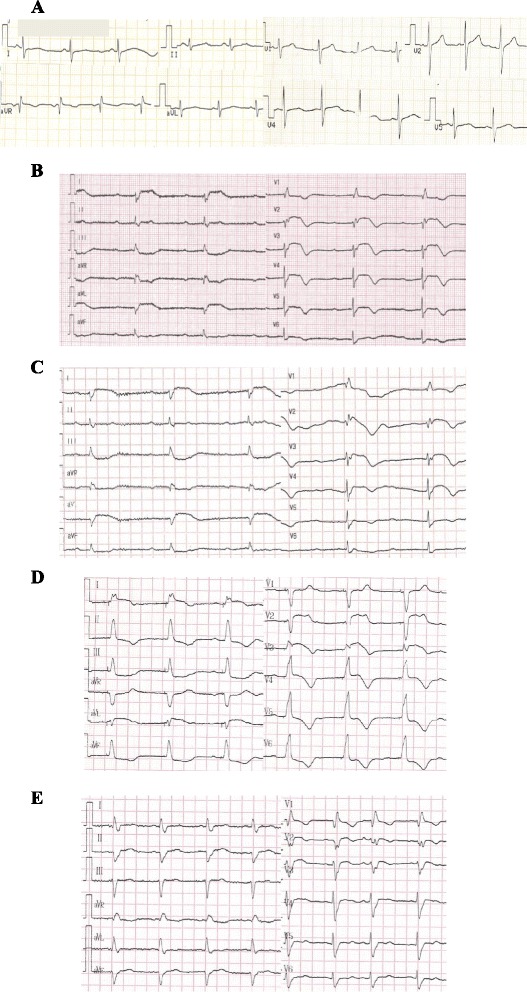
Electrocardiography development. **a** Almost normal ECG shows no conduction block. **b** ECG indicates first-degree AV block and CRBBB with precordial leads ST segment elevation. **c** ECG indicates third-degree AV block with precordial leads ST segment elevation. **d** ECG shows right ventricular stimulation of temporary pacemaker and elevated ST segment restoring (after emergency coronary angiography). **e** ECG indicates third-degree AV block disappeared and pacemaker removed, but CRBBB was left over

**Fig. 2:**
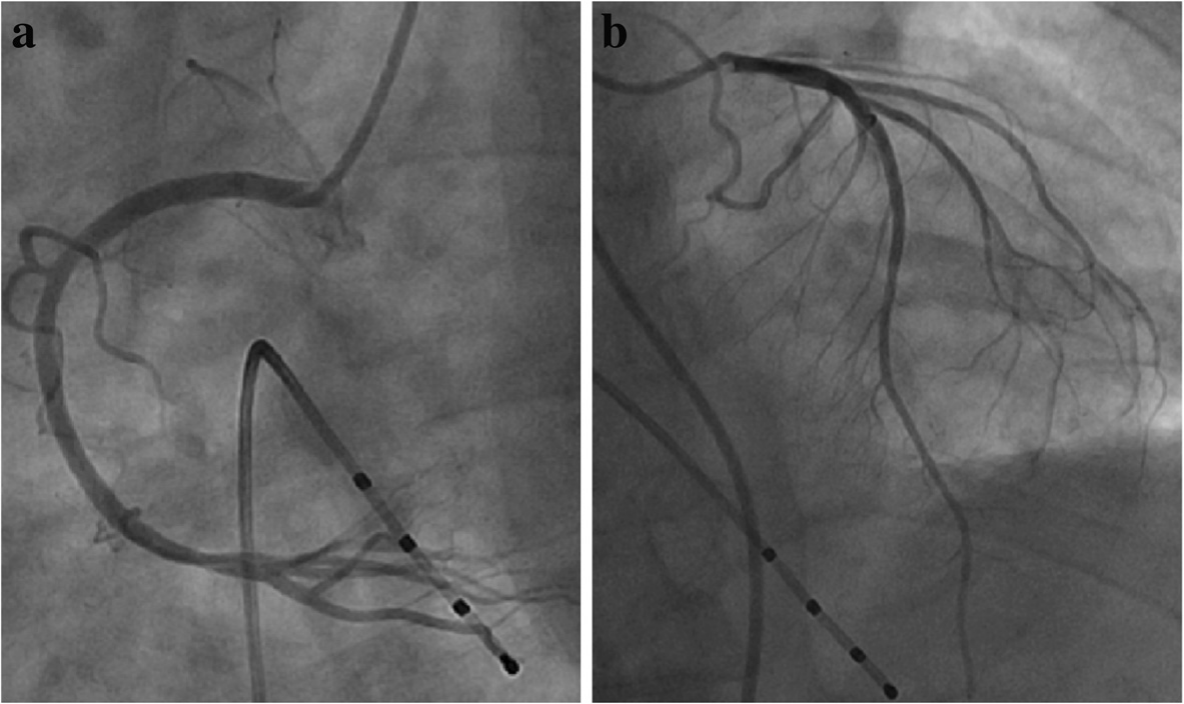
Coronary angiography. Emergency coronary angiography revealed no flow-limiting lesions. **a** For view of right coronary artery. **b** For view of left anterior descending artery and left circumflex artery

**Fig. 3:**
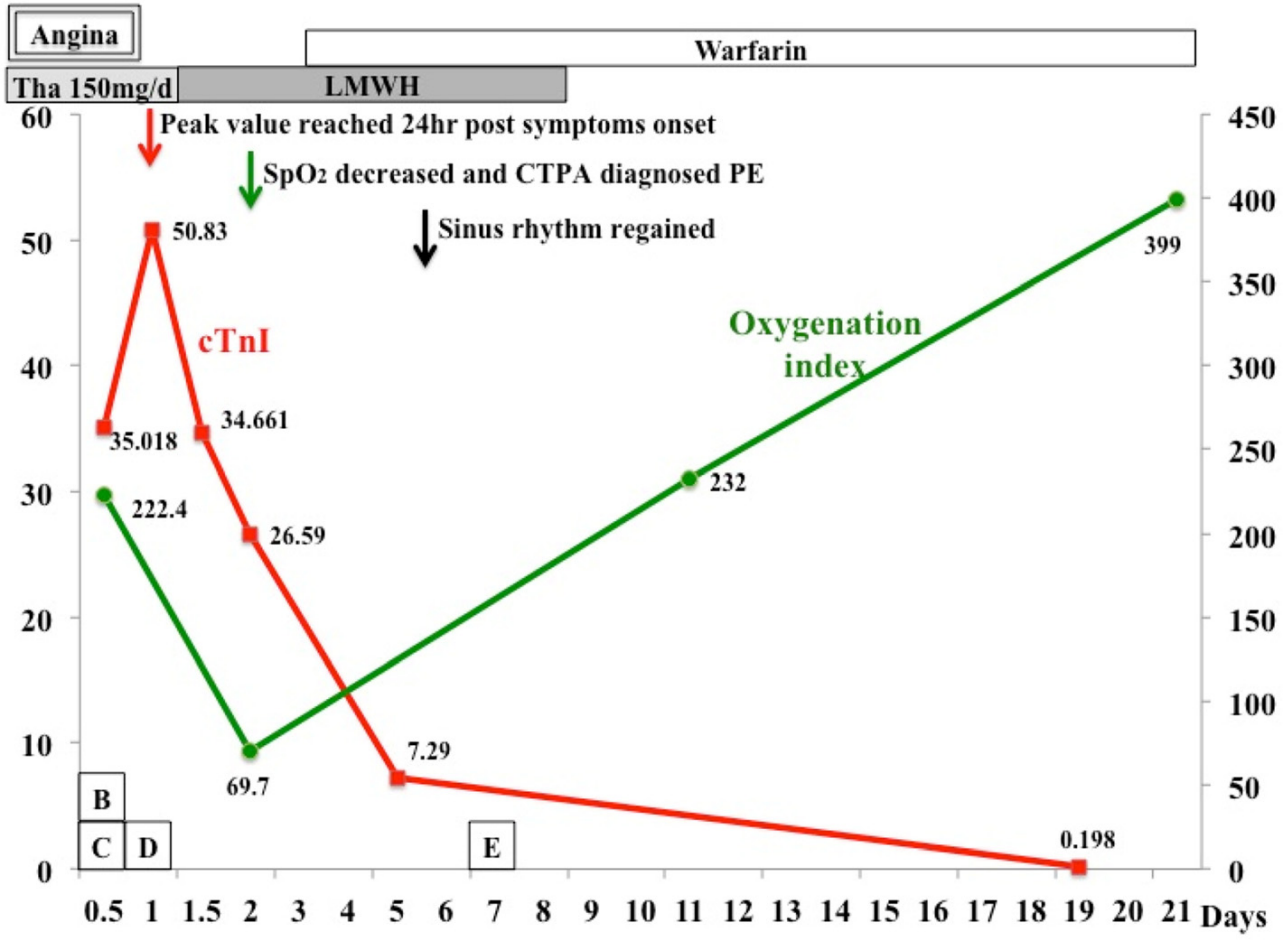
Clinical course. The solid red line indicates the cTnI level (μg/L). The solid green line indicates the oxygenation index (a ratio of the partial pressure of arterial oxygen to the fraction of inspired oxygen). Horizontal axis indicates time (days) after symptoms onset. *b*, *c*, *d* in textboxes indicates different ECG (for Fig. 1b, c, d and e, respectively). Tha = thalidomide. cTnI = cardiac troponin I

**Fig. 4:**
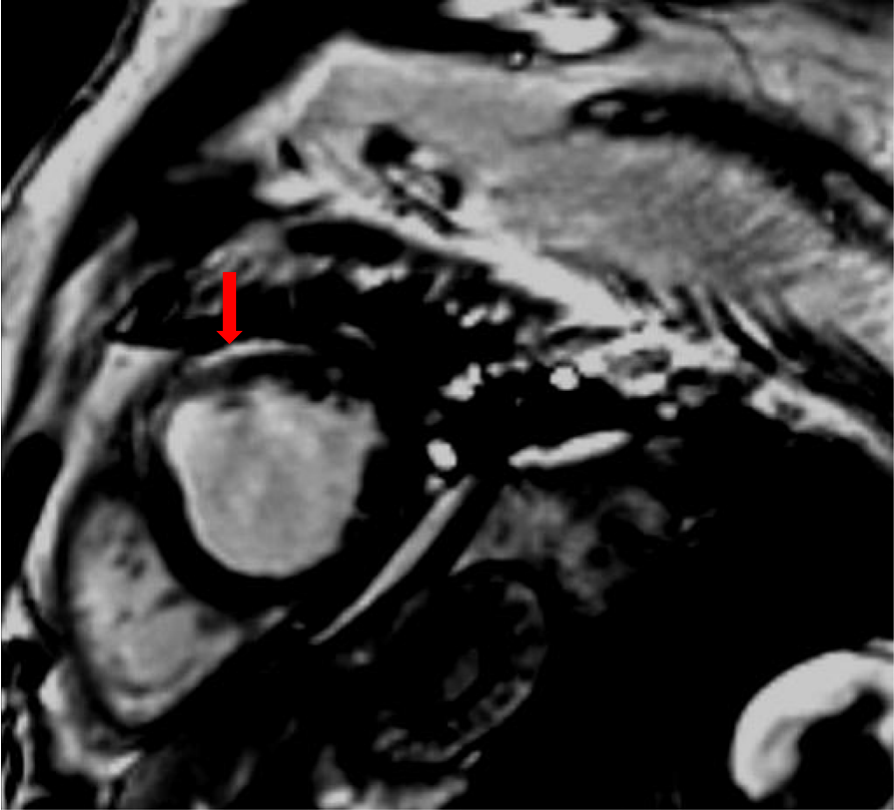
Cardiac magnetic resonance imaging. Cardiac MRI revealed thinning of apical anterior wall of left ventricle and subendocardial late gadolinium enhancement (*arrow*); estimated left ventricular EF was 44.3 %

**Fig. 5:**
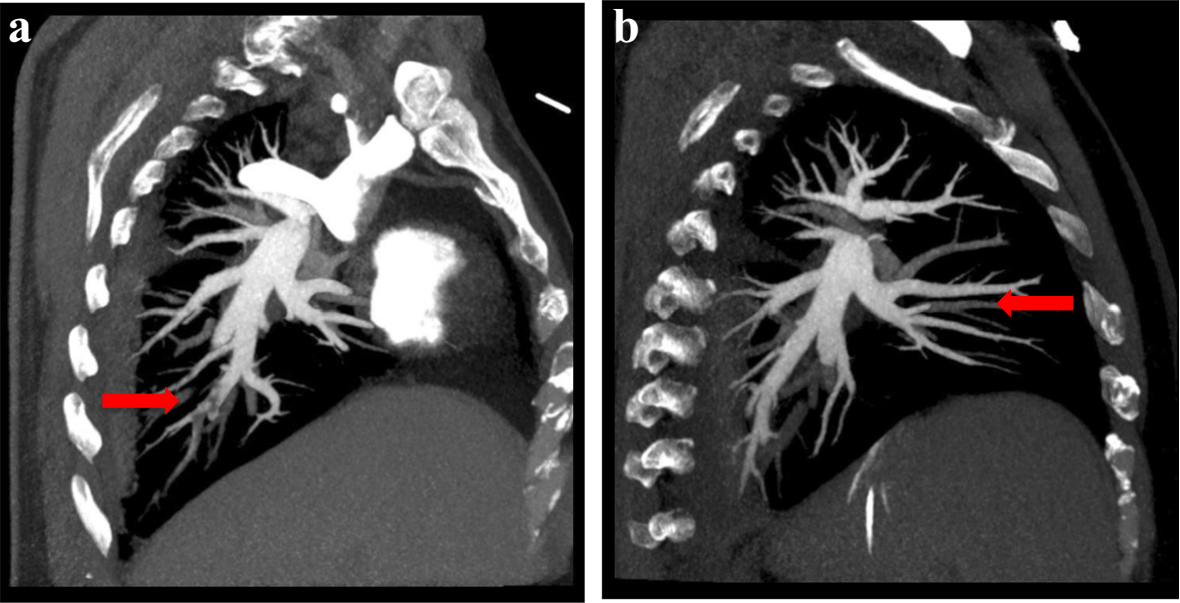
Computed tomography pulmonary angiography. CTPA revealed scatter embolism of subsegmental pulmonary arteries of both sides. **a**, **b** curved sheet reconstruction showed the emboli (*arrows*)

A 53-year old man presented in the emergency room because of suddenly onset of chest pain, nausea and then syncope for several minutes. Previous medical history included neurodermitis for which ebastine and ketotifen were given but proven ineffective, so thalidomide (150 mg/d) therapy was initiated 2 weeks before; medical history also included hypercholesterolemia and simvastatin taking. No other medication including corticosteroids was reported and electrocardiography (ECG) in the past was almost normal (Fig. 1a). The patient did not exhibit any other established risk factors for coronary artery disease, such as obesity, diabetes, hypertension or family history, with the exception of current smoking. Physical examination on admission revealed a heart rate of 47 bpm, blood pressure of 96/61 mmHg and normal SpO_2_ (98 % without oxygen). The 12-lead ECG showed sinus rhythm with a first-degree AV block and complete right bundle branch block (CRBBB), and I, aVL, V2-4 leads ST segment elevation (Fig. 1b); about 1 h later repeated ECG showed third-degree AV block but no ST-T dynamic change (Fig. 1c). So the diagnosis of acute coronary syndrome was suspected. The treatment of aspirin and clopidogrel was initiated for anti-platelet after which patient’s chest pain relieved partially, but intravenous nitroglycerin was avoided because of relatively low blood pressure and slow heart rate. 1 h after presentation, emergency coronary angiography was conducted and demonstrated no flow-limiting lesions in coronary arteries except minor atherosclerosis (Fig. 2), and meanwhile temporary pacemaker was implanted (Fig. 1d). Hours later the patient was admitted into cardiac care unit, blood tests showed LDL-C level 2.23 mmol/L and cardiac troponin I (cTnI) level elevated significantly (maximum 50.83 μg/L reached at 24 h after symptoms onset, normal upper limit was 0.04 μg/L; Fig. 3). And echocardiography only showed hypokinesis of middle and inferior segment of anterior wall with normal ejaculation fraction (EF) of 53 %, without tricuspid regurgitation or pulmonary hypertension, and no evidence of left or right-sided heart thrombi or right-left shunt was found; ^99m^Tc-MIBI myocardial perfusion single photon emission computed tomography showed reduced perfusion in the middle anterior wall of left ventricle; and further cardiac magnetic resonance imaging (MRI) showed thinning of apical anterior wall of left ventricle and subendocardial late gadolinium enhancement (Fig. 4). So it was likely that acute coronary event had happened. In CCU we continued aspirin for anti-platelet, but 2 days later patient’s SpO_2_ fell to 95 % at 10 L/min oxygen with reservoir mask, and D-dimer level elevated significantly (3.06 mg/L, normal range within 0.55 mg/L). Further ultrasound examination found no thrombi in deep veins of both lower limbs, but computed tomography pulmonary angiography (CTPA) revealed scattered embolism of subsegmental pulmonary arteries of both sides (Fig. 5). Tests for hypercoagulability was ordered and demonstrated normal coagulation parameters (also including factor V Leiden mutation and levels of protein C, protein S and anti-thrombin III), normal homocysteine level, negative anti-nuclear antibodies, anti-cardiolipin antibodies, lupus anticoagulant and tumor markers. So thalidomide was thought to be the cause of hypercoagulability, and this medication was ceased immediately; therapeutic low molecule weight heparin was initiated and then switched to warfarin with a target INR of 2 ~ 3, and nifedipine was given for coronary spasm. The patient reported no symptoms after therapy modification and SpO_2_ rose up to normal (97 % without oxygen) gradually, and third degree AV block also disappeared during hospitalization with temporary pacemaker removed (Fig. 1e). After discharge, patient was followed up over a period of 12 months, and warfarin was continued for 3 months with appropriate INR. No further recurrences of chronotropic or ischemic cardiac symptoms were reported.

## Discussion

The severe teratogenic side effects of thalidomide led to its well-publicized withdrawal in the 1970s. Thalidomide appears to have several immunomodulatory properties, including suppression of TNF-α production, down-regulation of cell-surface adhesion molecules involved in leukocyte migration, decreasing circulating helper T cell to suppressor T-cell ratio, and inhibition of IFN-γ, and more roles recognized including inhibition of angiogenesis as well as anti-fibrotic and anti-oncogenic properties [6]. Therefore thalidomide is being used in an increasing number of diseases nowadays.

Thalidomide is associated with higher risk of thromboembolism, both venous and arterial. Potential mechanisms have been explored: serum levels of the anticoagulant pathway cofactor thrombomodulin transiently dropped during the first month of thalidomide therapy, with gradual recovery over the following two months [7]; patients with MM treated with thalidomide had extremely high levels of von Willebrand factor antigen and factor VIII, factors known to be associated with an increased risk of thrombotic events in the general population [8]. But the reason for thromboembolism in the non-MM setting remains undefined. As noted in our patient, MI and PE had developed almost simultaneously. A reasonable explanation for PE is hypercoagulability induced by thalidomide caused grand thrombotic events in venous system. Because no right-left shunt was found in heart, paradoxical embolism of coronary artery from venous thrombi was not likely. Because coronary angiography demonstrated no flow-limiting lesions or thrombus in major coronary arteries, it probably was the coronary spasm that caused myocardial infarction [9], which had been reported as a rare complication of thalidomide therapy (only one case in the past) [2] and maybe further ergonovine provocation test was needed to ensure the existence of it. The precise mechanism that caused spasm remains undefined, and someone believed thalidomide could cause vasoconstriction through endothelial disruption [2]. And the ST segment elevation was persistent even though no obvious coronary spasm observed during angiography, probably because of acute myocardial infarction (likely ST elevated myocardial infarction) caused by coronary spasm. Another possible cause for MI was thrombus formed in coronary artery and needed to be confirmed by coronary angiography. Often thrombosis is formed at atherosclerotic site and seen during angiography, but may resolve spontaneously leaving almost no residual lesions. So this cause could not be completely ruled out.

Meanwhile Takotsubo cardiomyopathy was also considered as an important differential diagnosis, for chest pain symptoms, precordial leads ST segment elevation and negative result of coronary angiography. But we believed that Takotsubo cardiomyopathy stood a less possibility. The reasons were as follows: first, there was no risk factors such like stress trigger, sepsis or pheochromocytoma; second, cTnI elevated so obviously (above 1, 000 multiple of normal upper limit) [10] and dropped slowly, and the ratio between peak cTnI and EF measured at admission was 95.9 (much higher than cut-off value of ≤60) [11]; third, cardiac MRI showed significant late gadolinium enhancement of subendocardial area in anterior wall [12, 13]. But further follow-up was needed (such as echocardiography and cardiac MRI) to provide more evidence.

Cardiac adverse effects associated with thalidomide also include bradycardia [2–4]. The mechanism for this remains unclear although interference with autonomic nervous system has been suggested, with parasympathetic over activity caused by inhibition of TNF-α by thalidomide in the dorsal motor neurons of valgus nerve [4]. Despite a few reports of thalidomide-induced bradycardia, only one case of total AV-block have been published so far. Hinterseer M et al. [5] had reported the first case taking thalidomide developed third degree AV block, and thalidomide was ceased immediately but ECG didn’t regain sinus rhythm until 2 months later during which a permanent pacemaker was implanted. Our patient’s ECG also didn’t regained sinus rhythm until 3 days after thalidomide discontinuation. The mechanisms of additional long lasting effects of thalidomide (half-life period 4–7 h) on conduction system are not clear, but may base on stimulation of immune responses similar to myocarditis. It also informs us that if bradycardia or high grade AV block happens in patients taking thalidomide, maybe the ECG will be monitored for several days even up to a week before the decision of permanent pacemaker implantation.

Since high risk of thromboembolism, there are guidelines issued for antithrombotic prophylaxis in cancer clinical setting for thalidomide use [14]. Because there seems to be a similar and well-documented risk in the non-cancer setting, antithrombotic prophylaxis may be also effective and beneficial, but needs further investigation for optimal drug and dosage.

It is also important to monitor patients taking thalidomide for signs and symptoms of bradycardia or higher degree AV block, especially in those with concomitant medications affecting cardiac conduction system [3]. Early detection of conduction delay might help to modify therapy before symptomatic clinical events occur.

## Conclusion

This is the very first case in which myocardial infarction, third degree atrioventricular block and pulmonary embolism almost simultaneously developed. We should be ware that anti-thrombotic prophylaxis, which needs further investigation for optimal drug and dosage, may be beneficial in thalidomide therapy. And it is also important to monitor patients taking thalidomide for signs and symptoms of bradycardia or higher degree atrioventricular block.

## Consent

Written informed consent was obtained from the patient for publication of this Case report and any accompanying images. A copy of the written consent is available for review by the Editor of this journal.

## Abbreviations

AVB: atrioventricular block
CRBBB: complete right bundle branch block
cTnI: cardiac troponin I
CTPA: computed tomography pulmonary angiography
DVT: deep venous thrombus
ECG: electrocardiography
EF: ejaculation fraction
MI: myocardial infarction
MM: multiple myeloma
MRI: magnetic resonance imaging
PE: pulmonary embolism
Tha: thalidomide
